# The association of vascular disorders with incident dementia in different age groups

**DOI:** 10.1186/s13195-019-0496-x

**Published:** 2019-05-17

**Authors:** Nienke Legdeur, Sven J. van der Lee, Marcel de Wilde, Johan van der Lei, Majon Muller, Andrea B. Maier, Pieter Jelle Visser

**Affiliations:** 1 Alzheimer Center Amsterdam, Department of Neurology, Amsterdam Neuroscience, Vrije Universiteit Amsterdam, Amsterdam UMC, PO Box 7057, 1007 MB Amsterdam, the Netherlands; 2 Institute of Medical Informatics, Erasmus University Rotterdam, Rotterdam, the Netherlands; 3 Department of Internal Medicine, Amsterdam UMC, Amsterdam, the Netherlands; 4 Department of Medicine and Aged Care, @AgeMelbourne, Royal Melbourne Hospital, University of Melbourne, Melbourne, Australia; 5 Department of Human Movement Sciences, @AgeAmsterdam, Research Institute Amsterdam Movement Sciences, Vrije Universiteit Amsterdam, Amsterdam, the Netherlands; 6 Department of Psychiatry & Neuropsychology, School for Mental Health and Neuroscience, Maastricht University, Maastricht, the Netherlands

**Keywords:** Dementia, Primary care, Vascular disorders, Vascular disease, Cardiovascular risk factors, Aging

## Abstract

**Background:**

There is increasing evidence that dementia risk associated with vascular disorders is age dependent. Large population-based studies of incident dementia are necessary to further elucidate this effect. Therefore, the aim of the present study was to determine the association of vascular disorders with incident dementia in different age groups in a large primary care database.

**Methods:**

We included 442,428 individuals without dementia aged ≥ 65 years from the longitudinal primary care Integrated Primary Care Information (IPCI) database. We determined in 6 age groups (from 65–70 to ≥ 90 years) the risk of hypertension, diabetes mellitus, dyslipidemia, stroke, myocardial infarction, heart failure, and atrial fibrillation for all-cause dementia using incidence rate ratios, Cox regression, and Fine and Gray regression models.

**Results:**

The mean age at inclusion of the total study sample was 72.4 years, 45.7% of the participants were male, and median follow-up was 3.6 years. During 1.4 million person-years of follow-up, 13,511 individuals were diagnosed with dementia. The risk for dementia decreased with increasing age for all risk factors and was no longer significant in individuals aged ≥ 90 years. Adjusting for mortality as a competing risk did not change the results.

**Conclusions:**

We conclude that vascular disorders are no longer a risk factor for dementia at high age. Possible explanations include selective survival of individuals who are less susceptible to the negative consequences of vascular disorders and differences in follow-up time between individuals with and without a vascular disorder. Future research should focus on the identification of other risk factors than vascular disorders, for example, genetic or inflammatory processes, that can potentially explain the strong age-related increase in dementia risk.

**Electronic supplementary material:**

The online version of this article (10.1186/s13195-019-0496-x) contains supplementary material, which is available to authorized users.

## Background

Dementia is a major public health care problem with 35.6 million cases worldwide in 2010 [1]. Knowledge about the risk factors for dementia is essential to control the increasing number of patients with dementia [2]. Vascular disorders, such as hypertension and diabetes mellitus (DM), are modifiable risk factors for dementia [3–5], and it has been suggested that a substantial part of dementia cases could be prevented if these factors would be eliminated [6]. Both the incidence of dementia and the burden of vascular disorders are strongly age-dependent [7, 8]. On the one hand, neuropathological studies have shown that at high age, the majority of dementia cases have mixed pathologies with both neurodegeneration and cerebrovascular disease contributing to the cognitive impairment [9–12]. On the other hand, there is emerging evidence that the risk of dementia associated with cardiovascular risk factors decreases with age [3]. For example, hypertension was a risk factor for dementia when present during mid-life but became protective in individuals aged 80 years and older [13]. These conflicting findings between the risk factor and pathological studies remain poorly understood. Longitudinal population-based studies are needed to further elucidate the influence of age on the association between vascular disorders and dementia [14].

Databases from general practitioners (GPs) in the Netherlands are in particular suitable to study the association between vascular disorders and incident dementia. They contain a large and unbiased selection of the population with complete follow-up as citizens need to be registered with a GP practice, and there is an obligatory reporting of diagnoses from secondary care to GPs [15]. In the Netherlands, the Integrated Primary Care Information (IPCI) database contains a large number of electronic patient records from different GPs [15], which has been successfully used to study dementia previously [16].

In the present study, we used the IPCI database to elucidate the association of vascular disorders with incident dementia in groups of increasing age ranging from young-old (≥ 65 to 70 years) to oldest-old (≥ 90 years) individuals.

## Methods

### Study sample

The IPCI database is a research database with electronic patient records from GPs in the Netherlands [15]. It includes data from over two million individuals. The present study was approved by the Scientific and Ethical Advisory Board of the IPCI project (project number: 2015-14).

We included individuals aged ≥ 65 years at any time between the start of the IPCI database in January 1996 and January 2017. To enhance the reliability of the data, follow-up time did not include the first year a GP or patient is part of the IPCI database. The decision regarding the start age of inclusion was based on the positive predictive value of the incident dementia diagnosis in IPCI (Additional file 1: Table S1). All individuals with a dementia diagnosis at the age of 65 years or at study entry were excluded. Incident dementia was studied in six age groups: 65 to 70 years, 70 to 75 years, 75 to 80 years, 80 to 85 years, 85 to 90 years, and ≥ 90 years. Individuals with follow-up time spanning two or more age groups were included in all age groups to which they contributed follow-up time; hence, the sum of the individuals in all age groups exceeds the total number in the study sample.

### Study outcome

All-cause dementia was based on the International Classification of Primary Care version 1 (ICPC-1) code P70 determined by the GP or the use of anti-dementia drugs according to the Anatomical Therapeutic Chemical (ATC) classification system (ATC code: N06D which include anticholinesterases, memantine, Ginkgo folium, and combinations) [17]. The date of dementia diagnosis was based on the first ICPC or ATC code record in the database.

### Risk factors

We assessed seven vascular disorders as risk factors for incident dementia: hypertension, DM, dyslipidemia, stroke, myocardial infarction, heart failure, and atrial fibrillation (AF). Hypertension was based on ICPC code K86 or K87 or the use of antihypertensive medication (ATC code: C02, C03, C07, C08, C09). DM was based on ICPC code T90 or the use of antidiabetic medication/insulin (ATC code: A10). Dyslipidemia was based on ICPC code T93 or the use of statins (ATC code: C10). Stroke, ICPC code: K90; myocardial infarction, ICPC code: K75; heart failure, ICPC code: K77; and AF, ICPC code: K78, were based on ICPC code only. The dates of the risk factors were based on the first ICPC or ATC code records in the database. For every age group, the risk factor was defined as present when an individual had the risk factor at the start of that age group or at study entry.

### Statistical analyses

We estimated the risk for dementia of all seven vascular disorders within a 5-year period (except for individuals ≥ 90, in which no upper age limit was used) in two ways. We calculated the incidence rates and incidence rate ratios (IRR) of dementia per 1000 person-years (PY) follow-up to show the exact numbers of dementia cases per vascular disorder and age group and fitted Cox regression models with follow-up time as time scale, adjusting for age at study entry and sex (model 1, main model). These analyses were performed within each age group separately. We used follow-up time as the time scale, instead of age, because of the unequal baseline age distributions at the entry for the different age groups and the preference for follow-up time as time scale when the true time scale is uncertain [18, 19].

To test whether the effect of the vascular disorder on dementia risk was dependent on age, we added an interaction term between the vascular disorder and age group to the main Cox regression model in the total sample (further referred to as trend analyses). If the effect estimate of this interaction term is positive, it indicates that the effect of the vascular disorder on dementia risk increases with age. If the effect estimate of the interaction term is negative, it indicates that the effect of vascular disorder on dementia risk decreases with age.

In secondary analyses, Fine and Gray regression models were used to additionally adjust for death as a competing risk (model 2) [20], and the main Cox regression model was extended by additional adjustments for the other vascular disorders (model 3) and medication use (model 4). In model 4, we corrected for antihypertensive medication, antidiabetic medication/insulin, statins, loop diuretics (ATC code: B03C), vitamin K antagonists/direct factor Xa inhibitors (ATC code: B01AA/F), and platelet aggregation inhibitors (ATC code: B01AC). In models 1, 2, 3, and 4, individuals without the specific vascular disorder were used as the reference group. Only in model 5 we repeated Cox regression model 1 with individuals without any vascular disorder as the reference group.

In sensitivity analyses, we repeated model 1 (Cox regression model adjusted for age at study entry and sex) using age, instead of follow-up time, as time scale (model 6). Additionally, we repeated Cox and Fine and Gray regression models without limitation of follow-up time at the end of each age group (explanation of the limited and continuous follow-up time in Additional file 1: Figure S1). Limitation of the follow-up time as performed in the analyses described above might lead to an underestimation of the effect of a vascular disorder on dementia risk, and this effect might differ per age group. To test whether the limitation of follow-up time affected our results, we repeated the analyses with continuous follow-up time.

For the interpretation of our results, we also calculated the limited follow-up time difference between individuals with and without a vascular disorder.

Last, we additionally repeated the Cox regression model 1 (adjusted for age at study entry and sex) with mortality as the outcome to test per age group whether the vascular disorder was associated with mortality. We also repeated the trend analyses with mortality as the outcome to test whether the effect of the vascular disorder on mortality was dependent on age by adding an interaction term between the vascular disorder and age group to the Cox regression model.

The trend analyses for the seven vascular disorders were corrected for multiple testing using Bonferroni, defining the *p* value threshold for significance at < 0.0071 (= 0.05/7). For other analyses, we interpreted a *p* value < 0.05 as significant. R-Studio version 1.0.143 with R version 3.4.0 was used for all analyses [21]. “Rateratio.test” package was used to test IRR and the “survival” package for Cox regression analysis and the “cmprsk” package for the competing risk analyses [22–24].

## Results

**Fig. 1:**
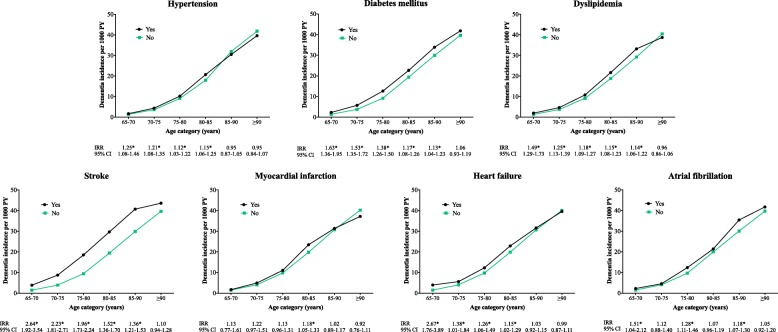
Association of risk factor with incident dementia per 5 year age groups (except for individuals ≥ 90, in which no upper age limit was used). IRR, incidence rate ratio; PY, person-years; CI, confidence interval; *p < 0.05

We included 442,428 individuals (45.7% male) with a mean age of 72.4 (SD 7.5, range 65.0–115.2) years at baseline. The median follow-up time of the total sample, without considering the limitations of the age groups, was 3.6 (range 0.0–10.7) years. During 1.4 million PY of follow-up, 13,511 individuals developed dementia. Incident dementia strongly increased with age from 1.5/1000 PY of follow-up at age 65–70 to 40.0/1000 PY of follow-up at age ≥ 90 (Table 1 and Fig. 1). Hypertension, DM, and dyslipidemia were the most prevalent risk factors with rates ranging from 14.8 to 84.4% (Table 1). The prevalence of all risk factors was higher in the older age groups, except for DM and dyslipidemia for which the prevalence was highest in individuals aged 80–85 years and lower in the following age groups (Table 1).

**Table 1:** Descriptive statistics ≥ 65-years-old individuals without dementia at study entry in IPCI

	65–70	70–75	75–80	80–85	85–90	≥ 90
Sample size, *N*	216828	167493	125971	89172	52739	23529
Follow-up time, median years (IQR)	1.92 (1.00–3.33)	1.84 (0.84–3.17)	1.86 (0.92–3.17)	1.83 (0.91–3.09)	1.67 (0.75–2.84)	1.50 (0.70–2.67)
Age at study entry or age at group entry, mean years (SD)	66.23 (1.58)	71.18 (1.56)	76.18 (1.55)	81.15 (1.54)	86.00 (1.44)	91.33 (2.24)
Male	49.16	47.89	45.28	41.23	35.74	28.59
Incident dementia (per 1000 PY)	1.52	4.10	9.88	20.09	30.76	40.00
Mortality rate per 1000 PY^a^	10.97	17.35	28.19	48.94	84.87	182.61
Hypertension prevalence	55.35	64.19	71.88	78.95	83.33	84.40
Diabetes mellitus prevalence	14.78	18.18	20.82	22.17	21.90	19.27
Dyslipidemia prevalence	37.99	44.29	47.35	47.09	41.55	29.84
Stroke prevalence	2.80	3.95	5.41	7.19	9.12	10.83
Myocardial infarction prevalence	4.07	5.25	6.30	7.41	7.70	7.76
Heart failure prevalence	1.64	2.77	5.02	8.70	14.05	21.14
Atrial fibrillation prevalence	3.57	5.63	8.03	11.22	14.73	17.14

Values are percentages unless stated otherwise; prevalence—the disease is present at the start of that age group or at database entry; individuals that contribute follow-up time to multiple age groups are included in all age groups they contribute to; ^a^calculated by dividing the total number of deceased individuals by the total number of person-years (PY) follow-up per 1000 in that specific age group. *IPCI* Interdisciplinary Processing of Clinical Information database, *IQR* interquartile range, *N* number, *SD* standard deviation

### Association of risk factors with incident dementia

**Fig. 2:**
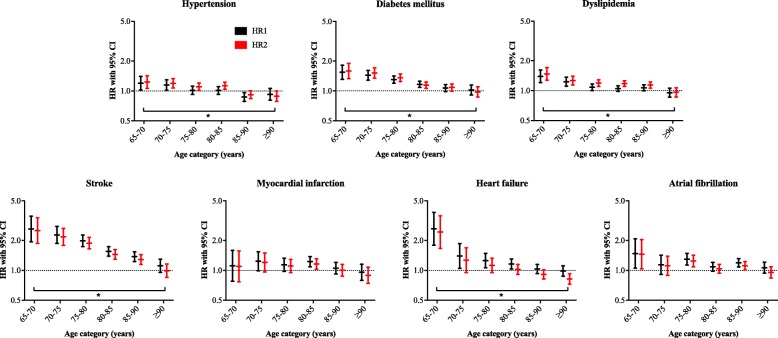
Risk of incident dementia in the presence of a risk factor per 5-year age group (except for individuals ≥ 90, in which no upper age limit was used). Hazard ratio from model 1 (HR1) determined with Cox regression analyses adjusted for age at study entry and sex; hazard ratio from model 2 (HR2) determined with competing risk analyses adjusted for age at study entry and sex; the HR on the *y*-axis is graphed on a log scale. CI, confidence interval; *HRs changed significantly (*p* < 0.0071) between age groups, determined with trend analysis including the interaction of age group with risk factor

The IRR of each risk factor as a function of age is shown in Fig. 1 and Additional file 1: Table S2. The hazard ratio (HR) for dementia of each risk factor as a function of age is shown in Fig. 2 and Additional file 1: Tables S3 and S4. The IRR and the HR of model 1 (HR1, corrected for age and sex) and model 2 (HR2, additional corrected for mortality) generally showed the same pattern with a decreased risk for dementia with increasing age. The trend analyses performed in model 1 showed that for all risk factors, except for dyslipidemia, there was a significant interaction between age group and the risk factor (Fig. 2, *p* values are reported below). This suggested that the incident dementia risk associated with these risk factors decreased with increasing age. We report below the HRs from model 1 (these are the same HRs as visualized in Fig. 2 and presented in Additional file 1: Table S3).

Hypertension was associated with an increased risk for incident dementia from age 65 to 85 years. The HR decreased from 1.24 (95% confidence interval (CI) 1.07–1.44) at age 65–70 years to 0.95 (95% CI 0.84–1.07) at age ≥ 90 years (*p* value for trend < 0.001). DM was associated with an increased risk for incident dementia from age 65 to 90 years. The HR decreased from 1.61 (95% CI 1.35–1.92) at age 65–70 years to 1.06 (95% CI 0.94–1.20) at age ≥ 90 years (*p* value for trend < 0.001). Dyslipidemia was associated with an increased risk for incident dementia from age 65 to 90 years. The HR decreased from 1.39 (95% CI 1.21–1.61) at age 65–70 years to 0.95 (95% CI 0.86–1.06) at age ≥ 90 years (*p* value for trend 0.11). Stroke was associated with incident dementia from age 65 to 90 years. The HR decreased from 2.61 (95% CI 1.94–3.51) at age 65–70 years to 1.11 (95% CI 0.95–1.30) at age ≥ 90 years (*p* value for trend < 0.001). Myocardial infarction was associated with incident dementia only in the individuals aged 80–85 years. The HR decreased from 1.11 (95% CI 0.78–1.59) at age 65–70 years to 0.96 (95% CI 0.79–1.15) at age ≥ 90 years (*p* value for trend 0.003). Heart failure was associated with incident dementia from age 65 to 85 years. The HR decreased from 2.62 (95% CI 1.80–3.83) at age 65–70 years to 0.99 (95% CI 0.87–1.11) at age ≥ 90 years (*p* value for trend < 0.001). AF was associated with incident dementia at age 65–70 years, age 75–80 years, and age 85–90 years. The HR decreased from 1.48 (95% CI 1.05–2.08) at age 65–70 years to 1.06 (95% CI 0.94–1.21) at age ≥ 90 years (*p* value for trend < 0.001).

Further correction for the other risk factors and medication use (models 3 and 4, Additional file 1: Table S4) yielded similar results, except that hypertension was no longer associated with incident dementia in individuals aged 65 to 85 years and that the HR related to hypertension did not decrease with age. Analyses with a control group without any risk factor as the reference (model 5, Additional file 1: Table S4) increased the HR, but the decrease of the HR with age was otherwise similar.

### Sensitivity analyses

Repeated analyses of model 1 using age instead of follow-up time as the time scale yielded similar results (model 6, Additional file 1: Table S5). This implicates that the time scale used for the present analyses does not substantially affect the results. Analyses without limitation of follow-up time at the end of each age group yielded similar results and showed the same pattern of decreasing HR with age (results not shown). This implicates that the pattern of decreasing HR with age cannot be explained by the limitation of follow-up time.

Follow-up was shorter in those with a risk factor than those without, with differences ranging from 1.1–3.0 months in the youngest age group to 2.7–4.2 months in the oldest age group (Additional file 1: Figure S2).

Analyses with mortality as the outcome showed that all risk factors were associated with an increased mortality risk in all age groups, except for dyslipidemia between the ages 80–85 and ≥ 90 years (Additional file 1: Table S6 ). Trend analyses indicated that mortality risk in the presence of a risk factor decreased with increasing age for all risk factors except hypertension (*p* value for trend for hypertension 0.35, for all other vascular disorders < 0.001).

## Discussion

The main finding is that the risk of vascular disorders for incident dementia decreases with age. Most vascular disorders were risk factors for dementia at age 65–70 years, but in individuals over 90 years, none of the vascular disorders was significantly associated with dementia. These findings implicate that prevention or treatment of vascular disorders might not be as effective to avert dementia in the oldest-old compared to younger populations [25] and reveal the persistent lack of our understanding of dementia in the oldest-old.

Age-dependent effects on incident dementia risk have been described for hypertension [3, 13, 26–28], dyslipidemia [3, 29], AF [4], and myocardial infarction [30] and a compound score including DM, hypertension, dyslipidemia, and myocardial infarction [31]. These studies found that these risk factors are associated with dementia in mid-life (age 40–65 years) but not in late-life (age > 65 years), with the exception for the compound score which was still associated with dementia in 70–79-year-old individuals but not in individuals over 80 years. For DM, an age-dependent effect (< 65 versus ≥ 65 years) on incident dementia was found [32], but an association of DM with dementia into late-life has also been reported [3, 33, 34]. For stroke, a decreasing dementia risk with increasing age (18 to ≥ 85 years) was reported [35, 36], but in these studies, stroke was still associated with incident dementia in individuals aged 85 years and older [13]. This might be explained by a difference in follow-up time, which was longer in these studies compared to the follow-up time in the oldest age group of our study, or differences in participant selection and study design. A direct comparison of the effect estimates over studies is difficult due to the differences in study design, adjustments for other risk factors or medication use, and the type of outcome studied (all-cause dementia versus vascular dementia or Alzheimer’s disease).

The age-dependent dementia risk can be explained by risk factor-specific explanations and explanations generalizable to all vascular disorders. High blood pressure might be necessary to secure cerebral blood flow at higher age [13, 27], and high cholesterol levels potentially reflect a better overall health status in older individuals [37]. Reverse causality might also be a possible explanation for the age-dependent effect of hypertension and dyslipidemia as blood pressure and cholesterol levels may drop before dementia onset as a consequence of the neurodegenerative process [38]. Possible explanations that are generalizable to all risk factors include the potential selective survival of individuals who are less susceptible to the negative consequences of the risk factors. Second, the higher prevalence of risk factors in older individuals dilutes the distinction between individuals with and without a risk factor. However, when we used individuals without any of the risk factors as a reference group, we observed a similar decrease of dementia risk with age.

The reduction could also be due to several biases associated with the risk factors. First, competing risk by mortality may be an explanation as individuals with a risk factor, such as hypertension, may die faster than those without the risk factor, and therefore are less likely to become demented [26]. However, adjustment for mortality as a competing risk did not change the findings and mortality risk in the presence of a risk factor did not increase with older age. Similarly, the presence of a risk factor may reduce the follow-up time in the IPCI database as individuals may be more likely, for example, to be reallocated to a permanent care facility. Follow-up was indeed shorter in those with a risk factor than those without, but differences were small and are unlikely to fully explain the difference.

Post-mortem neuropathological studies have indicated that with older age, other pathologies than AD also contribute to dementia risk such as TAR DNA-binding protein 43 (TDP-43) and hippocampal sclerosis [10–12]. Risk factors for non-AD pathology also include non-vascular risk factors, for example, autoimmune conditions and pro-inflammatory responses, such that the effect of vascular disorders on dementia risk in the oldest-old becomes attenuated [39, 40].

Another possible explanation may be the decrease in the prevalence of the apolipoprotein E (APOE ε4) allele, the major genetic risk factor for AD, with higher age [41]. The APOE ε4 allele seems to increase the risk for dementia in the presence of cardiovascular pathology, and a lower prevalence of the APOE4 allele could reduce the negative effect of vascular disorders on dementia incidence [42, 43].

However, it needs to be addressed that neuropathological studies in the oldest-old still report an important role for vascular diseases in dementia [10]. In vivo biomarker studies are necessary to provide more insight in this mismatch between the association of vascular markers with dementia in pathological studies and risk factor studies.

### Strengths and limitations

Previous studies typically reported age-dependent risks in mid-life (age 40–65 years) and late-life (age > 65 years), or only in old age (> 85 years) [3, 4, 13, 26, 29, 34]. Our findings are therefore unique as we tested the effect of age on risk factors for incident dementia in a population ranging from 65 to over 90 years. Other strengths and limitations of this study are mostly related to the characteristics of the IPCI database. The strength of IPCI is that it is a primary care database including a large number of individuals. Selection bias is limited as all citizens in the Netherlands need to be registered with a GP practice and the demographic characteristics of the individuals in IPCI are comparable with the overall Dutch population [44]. In addition, obligatory reporting of diagnoses from secondary care to GPs makes diagnoses more complete. The dementia incidence we observed in our study was comparable with other primary care or population-based cohort studies in the Netherlands [16, 45]. Limitations related to the IPCI database are that this database does not always contain the accurate starting date for the diagnoses as the GP regularly records date of subscription as the starting date for prevalent diagnoses. Therefore, we were not able to take the start date and duration of the vascular disorders into account. The second limitation is that no difference could be made between types of dementia as the ICPC coding system does not allow GPs to precisely record this. Also, GPs will not always refer their patients to perform extensive evaluations (neuropsychological testing and brain imaging) to determine the type of dementia. Third, GPs do not regularly measure blood pressure, glucose, or cholesterol levels in all their patients. Therefore, we could not take quantitative measurements into account. Fourth, as IPCI is an observational study, not all individuals were regularly evaluated by their GP. This could potentially lead to underreporting of the vascular disorders and dementia diagnoses. However, the prevalence of hypertension, DM, stroke, heart failure, and AF in IPCI is similar to that in population-based cohorts [45–51]. Still, the prevalence for myocardial infarction and dyslipidemia was lower in the IPCI database compared to population-based studies, which may suggest underdiagnoses, although the prevalence for dyslipidemia varies widely due to the differences in definition [52–54]. Fifth, follow-up time in IPCI ends when a GP changes software, and this limited the follow-up time in the present study. Sixth, we adjusted our analyses for age, sex, mortality, vascular disorders, and medication, but residual confounding is still possible as we were not able to correct for years of education and additional risk factors such as obesity and smoking. Finally, we tested only for a linear trend over the age groups and not for a non-linear trend. This might explain why the trend test for dyslipidemia was not significant.

## Conclusion

We found that the risk factors for dementia changed with age. All vascular disorders, except myocardial infarction, predicted incident dementia at age 65–70 years. The risk for dementia of these risk factors decreased with age, and none of them was predictive for dementia after the age of 90 years. Future research should focus on understanding the mismatch between the association of vascular markers with dementia in pathological studies and risk factor studies, in addition to unraveling other factors that could explain the age-related increase of dementia risk.

## Additional file

Additional file 1: **Table S1.** The positive predictive value (PPV) of the dementia diagnosis in IPCI per age group. **Table S2.** Incidence rate ratios (IRR) of dementia per age group according to risk factor. **Table S3.** Association of risk factors with incident dementia per age group. **Table S4.** Association of risk factors with incident dementia per age group (with additional adjustments). **Table S5.** Association of risk factors with incident dementia per age group using age as the time scale. **Table S6.** Association of risk factors with mortality per age group. **Figure S1.** Explanation of limited and continuous follow-up time. **Figure S2.** Follow-up (FU) time difference between individuals with and without a risk factor positive values mean a longer FU time in the individuals without a risk factor. (DOCX 260 kb)

## Abbreviations

AF: Atrial fibrillation
ATC: Anatomical Therapeutic Chemical
DM: Diabetes mellitus
GP: General practitioner
HR: Hazard ratio
ICPC-1: International Classification of Primary Care version 1
IPCI: Interdisciplinary Processing of Clinical Information
IRR: Incidence rate ratios
PY: Person-years
TDP-43: TAR DNA-binding protein 43
