# Supplementary material for: The association of vascular disorders with incident dementia in different age groups
Source: Alzheimers Res Ther. 2019 May 17;11:47. doi: 10.1186/s13195-019-0496-x (PMC6524321; doi:10.1186/s13195-019-0496-x)
Supplement: Supplementary file 1 — Table S1. The positive predictive value (PPV) of the dementia diagnosis in IPCI per age group. Table S2. Incidence rate ratios (IRR) of dementia per age group according to risk factor. Table S3. Association of risk factors with incident dementia per age group. Table S4. Association of risk factors with incident dementia per age group (with additional adjustments). Table S5. Association of risk factors with incident dementia per age group using age as the time scale. Table S6. Association of risk factors with mortality per age group. Figure S1. Explanation of limited and continuous follow-up time. Figure S2. Follow-up (FU) time difference between individuals with and without a risk factor positive values mean a longer FU time in the individuals without a risk factor. (DOCX 260 kb) [file 13195_2019_496_MOESM1_ESM.docx]

**Additional file 1:** Supplementary tables and figures

**Table S1** The positive predictive value (PPV) of the dementia diagnosis in IPCI per age group

| Age group (years) | Medical records checked (N) | Dementia diagnosis correct (N) | Dementia diagnosis incorrect (N) | Dementia diagnosis unclear (N) | PPV |
| --- | --- | --- | --- | --- | --- |
| ≥60-65 | 25 | 15 | 7 | 3 | 0.60 |
| ≥65-70 | 25 | 21 | 3 | 1 | 0.84 |
| ≥70-75 | 25 | 20 | 4 | 1 | 0.80 |
| ≥75-80 | 25 | 24 | 1 | 0 | 0.96 |
| ≥80-85 | 25 | 23 | 0 | 2 | 0.92 |
| ≥85-90 | 25 | 24 | 0 | 1 | 0.96 |
| ≥90 | 25 | 22 | 2 | 1 | 0.88 |

Medical records of 25 randomly selected individuals with a dementia diagnosis per five-year age range starting from the age of 60 years were checked by a medical doctor. The diagnosis of dementia was refuted if there were no other indications of a dementia diagnosis in the records apart from the ICPC-1 (International Classification of Primary Care version 1) code.

IPCI: Interdisciplinary Processing of Clinical Information database; N: number

**Table S2** Incidence Rate Ratios (IRR) of dementia per age group according to risk factor

| Risk factor | Age group | N dementia cases pos^a^ | PY pos^a^ | N dementia cases neg^b^ | PY neg^b^ | IRR (95% CI) | P-value |
| --- | --- | --- | --- | --- | --- | --- | --- |
| Hypertension | 65-70 | 440 | 262061.41 | 296 | 221215.00 | 1.25 (1.08-1.46)* | 2.70E-03 |
|  | 70-75 | 979 | 223857.81 | 481 | 132541.50 | 1.21 (1.08-1.35)* | 7.97E-04 |
|  | 75-80 | 1951 | 191155.38 | 723 | 79562.94 | 1.12 (1.03-1.22)* | 7.67E-03 |
|  | 80-85 | 2990 | 144440.01 | 737 | 41041.36 | 1.15 (1.06-1.25)* | 4.98E-04 |
|  | 85-90 | 2546 | 83452.77 | 571 | 17866.47 | 0.95 (0.87-1.05) | 3.27E-01 |
|  | ≥90 | 1475 | 37222.33 | 322 | 7697.64 | 0.95 (0.84-1.07) | 3.95E-01 |
| Diabetes Mellitus | 65-70 | 157 | 68981.74 | 579 | 414294.67 | 1.63 (1.36-1.95)* | 2.93E-07 |
|  | 70-75 | 362 | 63319.62 | 1098 | 293079.69 | 1.53 (1.35-1.72)* | 2.19E-11 |
|  | 75-80 | 680 | 53702.80 | 1994 | 217015.51 | 1.38 (1.26-1.5)* | 2.42E-12 |
|  | 80-85 | 892 | 39327.67 | 2835 | 146153.70 | 1.17 (1.08-1.26)* | 6.28E-05 |
|  | 85-90 | 706 | 20838.45 | 2411 | 80480.79 | 1.13 (1.04-1.23)* | 4.72E-03 |
|  | ≥90 | 327 | 7821.00 | 1470 | 37098.96 | 1.06 (0.93-1.19) | 3.96E-01 |
| Dyslipidemia | 65-70 | 339 | 175909.30 | 397 | 307367.11 | 1.49 (1.29-1.73)* | 9.78E-08 |
|  | 70-75 | 699 | 151006.97 | 761 | 205392.34 | 1.25 (1.13-1.39)* | 2.52E-05 |
|  | 75-80 | 1325 | 123021.68 | 1349 | 147696.63 | 1.18 (1.09-1.27)* | 2.24E-05 |
|  | 80-85 | 1820 | 84003.46 | 1907 | 101477.91 | 1.15 (1.08-1.23)* | 1.55E-05 |
|  | 85-90 | 1314 | 39592.03 | 1803 | 61727.21 | 1.14 (1.06-1.22)* | 4.81E-04 |
|  | ≥90 | 466 | 12037.56 | 1331 | 32882.41 | 0.96 (0.86-1.06) | 4.23E-01 |
| Stroke | 65-70 | 47 | 12192.69 | 689 | 471083.73 | 2.64 (1.92-3.54)* | 2.64E-08 |
|  | 70-75 | 108 | 12345.29 | 1352 | 344054.02 | 2.23 (1.81-2.71)* | 9.84E-13 |
|  | 75-80 | 238 | 12847.00 | 2436 | 257871.32 | 1.96 (1.71-2.24)* | 1.65E-19 |
|  | 80-85 | 346 | 11665.64 | 3381 | 173815.73 | 1.52 (1.36-1.70)* | 1.91E-12 |
|  | 85-90 | 330 | 8105.24 | 2787 | 93214.00 | 1.36 (1.21-1.53)* | 3.94E-07 |
|  | ≥90 | 182 | 4175.32 | 1615 | 40744.65 | 1.10 (0.94-1.28) | 2.41E-01 |
| Myocardial infarction | 65-70 | 32 | 18725.06 | 704 | 464551.36 | 1.13 (0.77-1.61) | 5.56E-01 |
|  | 70-75 | 86 | 17446.49 | 1374 | 338952.82 | 1.22 (0.97-1.51) | 9.48E-02 |
|  | 75-80 | 177 | 16001.78 | 2497 | 254716.54 | 1.13 (0.96-1.31) | 1.34E-01 |
|  | 80-85 | 297 | 12656.39 | 3430 | 172824.98 | 1.18 (1.05-1.33)* | 7.28E-03 |
|  | 85-90 | 228 | 7269.63 | 2889 | 94049.62 | 1.02 (0.89-1.17) | 7.82E-01 |
|  | ≥90 | 118 | 3173.40 | 1679 | 41746.56 | 0.92 (0.76-1.11) | 4.40E-01 |
| Heart failure | 65-70 | 28 | 7063.10 | 708 | 476213.32 | 2.67 (1.76-3.89)* | 1.39E-05 |
|  | 70-75 | 48 | 8574.88 | 1412 | 347824.43 | 1.38 (1.01-1.84)* | 4.21E-02 |
|  | 75-80 | 145 | 11806.85 | 2529 | 258911.47 | 1.26 (1.06-1.49)* | 1.04E-02 |
|  | 80-85 | 317 | 13836.93 | 3410 | 171644.44 | 1.15 (1.02-1.29)* | 1.83E-02 |
|  | 85-90 | 390 | 12343.87 | 2727 | 88975.37 | 1.03 (0.92-1.15) | 5.90E-01 |
|  | ≥90 | 321 | 8110.09 | 1476 | 36809.87 | 0.99 (0.87-1.11) | 8.62E-01 |
| Atrial fibrillation | 65-70 | 35 | 15465.95 | 701 | 467810.46 | 1.51 (1.04-2.12)* | 2.94E-02 |
|  | 70-75 | 83 | 18239.62 | 1377 | 338159.69 | 1.12 (0.88-1.40) | 3.54E-01 |
|  | 75-80 | 238 | 19243.27 | 2436 | 251475.05 | 1.28 (1.11-1.46)* | 5.50E-04 |
|  | 80-85 | 401 | 18741.78 | 3326 | 166739.59 | 1.07 (0.96-1.19) | 1.95E-01 |
|  | 85-90 | 466 | 13150.04 | 2651 | 88169.20 | 1.18 (1.07-1.30)* | 1.42E-03 |
|  | ≥90 | 275 | 6589.08 | 1522 | 38330.88 | 1.05 (0.92-1.20) | 4.65E-01 |

^a^Number (N) of dementia cases and Person Years (PY) in the individuals with a risk factor; ^b^N of dementia cases and PY in the individuals without a risk factor. *P-value <0.05. Incident rates are plotted in Fig. 1 of the main manuscript.

**Table S3** Association of risk factors with incident dementia per age group

| Risk factor | Age group | HR1 (95% CI) | P-value | HR2 (95% CI) | P-value |
| --- | --- | --- | --- | --- | --- |
| Hypertension | 65-70 | 1.24 (1.07-1.44)* | 4.47E-03 | 1.23 (1.06-1.42)* | 6.40E-03 |
|  | 70-75 | 1.21 (1.08-1.35)* | 6.47E-04 | 1.19 (1.07-1.33)* | 1.50E-03 |
|  | 75-80 | 1.12 (1.03-1.23)* | 6.89E-03 | 1.10 (1.01-1.2)* | 2.30E-02 |
|  | 80-85 | 1.15 (1.06-1.25)* | 5.39E-04 | 1.13 (1.04-1.22)* | 3.50E-03 |
|  | 85-90 | 0.96 (0.87-1.05) | 3.34E-01 | 0.92 (0.84-1.00) | 5.70E-02 |
|  | ≥90 | 0.95 (0.84-1.07) | 4.25E-01 | 0.88 (0.78-1.00) | 4.60E-02 |
| Diabetes Mellitus | 65-70 | 1.61 (1.35-1.92)* | 1.40E-07 | 1.59 (1.33-1.89)* | 3.20E-07 |
|  | 70-75 | 1.54 (1.37-1.74)* | 9.95E-13 | 1.51 (1.34-1.71)* | 8.30E-12 |
|  | 75-80 | 1.39 (1.27-1.52)* | 1.13E-13 | 1.36 (1.24-1.48)* | 6.70E-12 |
|  | 80-85 | 1.18 (1.10-1.27)* | 1.49E-05 | 1.14 (1.06-1.23)* | 5.80E-04 |
|  | 85-90 | 1.14 (1.04-1.24)* | 2.81E-03 | 1.08 (1.00-1.18) | 6.50E-02 |
|  | ≥90 | 1.06 (0.94-1.20) | 3.13E-01 | 0.98 (0.87-1.10) | 6.90E-01 |
| Dyslipidemia | 65-70 | 1.39 (1.21-1.61)* | 1.03E-07 | 1.47 (1.27-1.70)* | 1.90E-07 |
|  | 70-75 | 1.23 (1.11-1.36)* | 4.05E-06 | 1.26 (1.14-1.40)* | 7.80E-06 |
|  | 75-80 | 1.08 (1.00-1.17)* | 2.03E-06 | 1.19 (1.11-1.29)* | 5.30E-06 |
|  | 80-85 | 1.05 (0.98-1.12)* | 2.01E-07 | 1.18 (1.10-1.25)* | 7.00E-07 |
|  | 85-90 | 1.07 (0.99-1.15)* | 5.09E-05 | 1.14 (1.06-1.22)* | 3.40E-04 |
|  | ≥90 | 0.95 (0.86-1.06) | 7.84E-01 | 0.96 (0.87-1.07) | 4.80E-01 |
| Stroke | 65-70 | 2.61 (1.94-3.51)* | 2.16E-10 | 2.52 (1.87-3.38)* | 9.70E-10 |
|  | 70-75 | 2.27 (1.87-2.77)* | 2.22E-16 | 2.17 (1.79-2.65)* | 7.80E-15 |
|  | 75-80 | 1.98 (1.74-2.27)* | <1.00E-16 | 1.88 (1.65-2.15)* | <1.00E-16 |
|  | 80-85 | 1.55 (1.39-1.73)* | 9.44E-15 | 1.45 (1.30-1.62)* | 5.00E-11 |
|  | 85-90 | 1.38 (1.23-1.54)* | 4.44E-08 | 1.29 (1.15-1.44)* | 1.60E-05 |
|  | ≥90 | 1.11 (0.95-1.30) | 1.75E-01 | 0.99 (0.85-1.16) | 9.30E-01 |
| Myocardial infarction | 65-70 | 1.11 (0.78-1.59) | 5.52E-01 | 1.10 (0.77-1.57) | 6.20E-01 |
|  | 70-75 | 1.24 (0.99-1.54) | 5.80E-02 | 1.20 (0.97-1.50) | 1.00E-01 |
|  | 75-80 | 1.14 (0.98-1.33) | 9.91E-02 | 1.11 (0.95-1.29) | 2.00E-01 |
|  | 80-85 | 1.22 (1.08-1.38)* | 9.84E-04 | 1.16 (1.03-1.31)* | 1.60E-02 |
|  | 85-90 | 1.05 (0.92-1.20) | 4.72E-01 | 1.00 (0.87-1.15) | 9.80E-01 |
|  | ≥90 | 0.96 (0.79-1.15) | 6.48E-01 | 0.89 (0.74-1.08) | 2.30E-01 |
| Heart failure | 65-70 | 2.62 (1.80-3.83)* | 5.73E-07 | 2.43 (1.67-3.54)* | 4.00E-06 |
|  | 70-75 | 1.39 (1.05-1.86)* | 2.38E-02 | 1.27 (0.95-1.69) | 1.10E-01 |
|  | 75-80 | 1.26 (1.06-1.48)* | 7.82E-03 | 1.12 (0.95-1.33) | 1.70E-01 |
|  | 80-85 | 1.16 (1.03-1.30)* | 1.22E-02 | 1.02 (0.91-1.15) | 7.10E-01 |
|  | 85-90 | 1.03 (0.93-1.15) | 5.58E-01 | 0.91 (0.82-1.02) | 9.40E-02 |
|  | ≥90 | 0.99 (0.87-1.11) | 8.32E-01 | 0.82 (0.73-0.92)* | 1.20E-03 |
| Atrial fibrillation | 65-70 | 1.48 (1.05-2.08)* | 2.46E-02 | 1.46 (1.04-2.04)* | 3.00E-02 |
|  | 70-75 | 1.14 (0.91-1.42) | 2.52E-01 | 1.11 (0.89-1.39) | 3.40E-01 |
|  | 75-80 | 1.29 (1.13-1.48)* | 1.52E-04 | 1.24 (1.09-1.42)* | 1.30E-03 |
|  | 80-85 | 1.09 (0.98-1.21) | 1.14E-01 | 1.04 (0.94-1.15) | 4.90E-01 |
|  | 85-90 | 1.19 (1.08-1.32)* | 4.62E-04 | 1.11 (1.01-1.23)* | 3.40E-02 |
|  | ≥90 | 1.06 (0.94-1.21) | 3.44E-01 | 0.95 (0.84-1.08) | 4.70E-01 |

Hazard Ratio from model 1 (HR1) determined with Cox regression analyses adjusted for age at study entry and sex; Hazard Ratio from model 2 (HR2) determined with competing risk analyses adjusted for age at study entry and sex. *P-value <0.05. HR1 and HR2 are plotted in Fig. 2 of the main manuscript.

**Table S4** Association of risk factors with incident dementia per age group (with additional adjustments)

| Risk factor | Age group | HR3 (95% CI) | P-value | HR4 (95% CI) | P-value | HR5 (95% CI) | P-value |
| --- | --- | --- | --- | --- | --- | --- | --- |
| Hypertension | 65-70 | 0.97 (0.82-1.15) | 7.42E-01 | 0.96 (0.81-1.14) | 6.41E-01 | 1.34 (1.14-1.57)* | 4.17E-04 |
|  | 70-75 | 1.03 (0.91-1.16) | 6.31E-01 | 0.97 (0.85-1.11) | 6.82E-01 | 1.29 (1.15-1.46)* | 3.05E-05 |
|  | 75-80 | 0.97 (0.88-1.06) | 4.81E-01 | 0.87 (0.78-0.96)* | 6.18E-03 | 1.20 (1.09-1.32)* | 2.23E-04 |
|  | 80-85 | 1.04 (0.95-1.13) | 3.77E-01 | 0.91 (0.83-1.00) | 5.53E-02 | 1.26 (1.15-1.38)* | 7.26E-07 |
|  | 85-90 | 0.86 (0.78-0.94)* | 1.80E-03 | 0.79 (0.71-0.87)* | 1.08E-05 | 1.01 (0.91-1.12) | 8.93E-01 |
|  | ≥90 | 0.94 (0.82-1.06) | 3.09E-01 | 0.91 (0.79-1.05) | 2.03E-01 | 0.97 (0.85-1.11) | 6.89E-01 |
| Diabetes Mellitus | 65-70 | 1.36 (1.12-1.65)* | 1.85E-03 | 1.37 (1.15-1.64)* | 5.51E-04 | 1.81 (1.47-2.22)* | 1.58E-08 |
|  | 70-75 | 1.44 (1.26-1.64)* | 3.75E-08 | 1.36 (1.20-1.53)* | 9.60E-07 | 1.72 (1.48-1.99)* | 5.17E-13 |
|  | 75-80 | 1.34 (1.22-1.47)* | 1.54E-09 | 1.24 (1.13-1.35)* | 2.24E-06 | 1.50 (1.34-1.68)* | 3.29E-12 |
|  | 80-85 | 1.12 (1.04-1.21)* | 4.92E-03 | 1.12 (1.04-1.20)* | 4.03E-03 | 1.39 (1.25-1.54)* | 2.48E-09 |
|  | 85-90 | 1.10 (1.01-1.20)* | 2.85E-02 | 1.04 (0.96-1.14) | 3.32E-01 | 1.12 (0.99-1.26) | 6.48E-02 |
|  | ≥90 | 1.08 (0.95-1.22) | 2.31E-01 | 1.04 (0.93-1.17) | 4.82E-01 | 1.03 (0.88-1.22) | 6.87E-01 |
| Dyslipidemia | 65-70 | 1.28 (1.08-1.52)* | 5.28E-03 | 1.17 (0.98-1.38) | 7.48E-02 | 1.54 (1.30-1.82)* | 5.82E-07 |
|  | 70-75 | 1.06 (0.93-1.19) | 3.82E-01 | 1.01 (0.90-1.14) | 8.66E-01 | 1.39 (1.22-1.57)* | 5.55E-07 |
|  | 75-80 | 1.05 (0.96-1.14) | 3.14E-01 | 0.91 (0.84-1.00)* | 4.11E-02 | 1.28 (1.15-1.41)* | 2.05E-06 |
|  | 80-85 | 1.08 (1.00-1.16)* | 3.73E-02 | 0.94 (0.87-1.01) | 7.53E-02 | 1.34 (1.22-1.47)* | 3.29E-09 |
|  | 85-90 | 1.13 (1.05-1.23)* | 1.85E-03 | 1.01 (0.94-1.10) | 7.44E-01 | 1.10 (0.99-1.23) | 7.58E-02 |
|  | ≥90 | 0.98 (0.87-1.09) | 6.70E-01 | 0.94 (0.84-1.05) | 2.67E-01 | 0.96 (0.82-1.13) | 6.48E-01 |
| Stroke | 65-70 | 2.23 (1.65-3.02)* | 2.20E-07 | 2.07 (1.52-2.82)* | 3.45E-06 | 3.05 (2.22-4.18)* | 4.63E-12 |
|  | 70-75 | 2.10 (1.72-2.57)* | 4.49E-13 | 1.96 (1.60-2.40)* | 8.26E-11 | 2.66 (2.14-3.31)* | <1.00E-16 |
|  | 75-80 | 1.89 (1.64-2.16)* | <1.00E-16 | 1.81 (1.57-2.07)* | <1.00E-16 | 2.22 (1.90-2.59)* | <1.00E-16 |
|  | 80-85 | 1.48 (1.33-1.66)* | 7.08E-12 | 1.46 (1.30-1.63)* | 9.80E-11 | 1.82 (1.59-2.09)* | <1.00E-16 |
|  | 85-90 | 1.34 (1.20-1.51)* | 6.51E-07 | 1.33 (1.18-1.49)* | 2.12E-06 | 1.36 (1.18-1.57)* | 2.99E-05 |
|  | ≥90 | 1.12 (0.96-1.31) | 1.44E-01 | 1.13 (0.96-1.32) | 1.31E-01 | 1.08 (0.89-1.31) | 4.30E-01 |
| Myocardial infarction | 65-70 | 0.87 (0.60-1.25) | 4.55E-01 | 0.74 (0.51-1.08) | 1.14E-01 | 1.37 (0.94-1.99) | 1.06E-01 |
|  | 70-75 | 1.11 (0.89-1.40) | 3.46E-01 | 0.97 (0.77-1.22) | 7.71E-01 | 1.52 (1.19-1.93)* | 6.88E-04 |
|  | 75-80 | 1.06 (0.91-1.25) | 4.36E-01 | 0.97 (0.82-1.14) | 6.78E-01 | 1.32 (1.11-1.57)* | 1.94E-03 |
|  | 80-85 | 1.14 (1.01-1.29)* | 3.23E-02 | 1.11 (0.98-1.25) | 1.17E-01 | 1.52 (1.31-1.76)* | 1.72E-08 |
|  | 85-90 | 1.00 (0.87-1.16) | 9.55E-01 | 0.98 (0.85-1.12) | 7.32E-01 | 1.06 (0.90-1.25) | 4.96E-01 |
|  | ≥90 | 0.97 (0.80-1.17) | 7.38E-01 | 0.97 (0.80-1.18) | 7.57E-01 | 0.94 (0.75-1.17) | 5.77E-01 |
| Heart failure | 65-70 | 2.14 (1.45-3.16)* | 1.41E-04 | 1.98 (1.33-2.94)* | 6.93E-04 | 3.11 (2.10-4.62)* | 1.61E-08 |
|  | 70-75 | 1.18 (0.88-1.59) | 2.64E-01 | 1.15 (0.86-1.55) | 3.55E-01 | 1.72 (1.27-2.33)* | 4.31E-04 |
|  | 75-80 | 1.11 (0.94-1.32) | 2.29E-01 | 1.10 (0.93-1.31) | 2.70E-01 | 1.45 (1.21-1.74)* | 7.63E-05 |
|  | 80-85 | 1.09 (0.96-1.22) | 1.75E-01 | 1.08 (0.96-1.22) | 1.90E-01 | 1.37 (1.19-1.58)* | 8.48E-06 |
|  | 85-90 | 0.99 (0.89-1.10) | 8.49E-01 | 0.99 (0.89-1.11) | 9.09E-01 | 1.04 (0.91-1.19) | 5.65E-01 |
|  | ≥90 | 0.98 (0.86-1.11) | 7.39E-01 | 0.99 (0.87-1.12) | 8.63E-01 | 0.97 (0.82-1.15) | 7.17E-01 |
| Atrial fibrillation | 65-70 | 1.23 (0.87-1.75) | 2.42E-01 | 1.16 (0.79-1.70) | 4.61E-01 | 1.76 (1.23-2.52)* | 2.00E-03 |
|  | 70-75 | 1.02 (0.81-1.28) | 8.50E-01 | 0.99 (0.77-1.28) | 9.55E-01 | 1.40 (1.10-1.78)* | 6.34E-03 |
|  | 75-80 | 1.21 (1.06-1.39)* | 5.53E-03 | 1.18 (1.01-1.38)* | 3.22E-02 | 1.48 (1.27-1.72)* | 6.57E-07 |
|  | 80-85 | 1.02 (0.92-1.14) | 6.51E-01 | 1.02 (0.91-1.16) | 6.99E-01 | 1.32 (1.16-1.50)* | 2.87E-05 |
|  | 85-90 | 1.20 (1.08-1.32)* | 5.80E-04 | 1.19 (1.06-1.34)* | 3.74E-03 | 1.18 (1.04-1.35)* | 1.27E-02 |
|  | ≥90 | 1.07 (0.94-1.22) | 3.00E-01 | 1.08 (0.93-1.26) | 2.97E-01 | 1.03 (0.86-1.22) | 7.64E-01 |

Hazard Ratio from model 3 (HR3) determined with Cox regression analyses adjusted for age at study entry, sex and the other risk factors; Hazard Ratio from model 4 (HR4) determined with Cox regression analyses adjusted for age at study entry, sex and medication use; Hazard Ratio from model 5 (HR5) determined with Cox regression analyses adjusted for age at study entry, sex and individuals without any risk factor as reference group. *P-value <0.05.

**Table S5** Association of risk factors with incident dementia per age group using age as time scale

| Risk factor | Age group | HR6 (95% CI) | P-value |
| --- | --- | --- | --- |
| Hypertension | 65-70 | 1.25 (1.07-1.45)* | 3.56E-03 |
|  | 70-75 | 1.18 (1.06-1.32)* | 3.10E-03 |
|  | 75-80 | 1.12 (1.03-1.22)* | 9.67E-03 |
|  | 80-85 | 1.13 (1.04-1.23)* | 2.97E-03 |
|  | 85-90 | 0.95 (0.86-1.04) | 2.58E-01 |
|  | ≥90 | 0.96 (0.84-1.09) | 5.51E-01 |
| Diabetes Mellitus | 65-70 | 1.61 (1.35-1.92)* | 1.33E-07 |
|  | 70-75 | 1.51 (1.34-1.70)* | 8.41E-12 |
|  | 75-80 | 1.36 (1.25-1.49)* | 3.09E-12 |
|  | 80-85 | 1.17 (1.09-1.27)* | 2.89E-05 |
|  | 85-90 | 1.14 (1.05-1.24)* | 2.72E-03 |
|  | ≥90 | 1.09 (0.96-1.22) | 1.77E-01 |
| Dyslipidemia | 65-70 | 1.50 (1.29-1.73)* | 5.73E-08 |
|  | 70-75 | 1.25 (1.13-1.39)* | 1.97E-05 |
|  | 75-80 | 1.19 (1.11-1.29)* | 5.29E-06 |
|  | 80-85 | 1.19 (1.12-1.27)* | 6.40E-08 |
|  | 85-90 | 1.18 (1.10-1.27)* | 6.58E-06 |
|  | ≥90 | 1.03 (0.92-1.14) | 6.38E-01 |
| Stroke | 65-70 | 2.62 (1.95-3.52)* | 1.77E-10 |
|  | 70-75 | 2.18 (1.79-2.65)* | 7.44E-15 |
|  | 75-80 | 1.92 (1.68-2.19)* | <1.00E-16 |
|  | 80-85 | 1.50 (1.35-1.68)* | 5.58E-13 |
|  | 85-90 | 1.34 (1.20-1.51)* | 4.24E-07 |
|  | ≥90 | 1.10 (0.95-1.29) | 2.12E-01 |
| Myocardial infarction | 65-70 | 1.11 (0.78-1.59) | 5.53E-01 |
|  | 70-75 | 1.21 (0.97-1.51) | 8.98E-02 |
|  | 75-80 | 1.12 (0.96-1.30) | 1.52E-01 |
|  | 80-85 | 1.21 (1.07-1.36)* | 1.95E-03 |
|  | 85-90 | 1.04 (0.91-1.20) | 5.40E-01 |
|  | ≥90 | 0.94 (0.78-1.14) | 5.29E-01 |
| Heart failure | 65-70 | 2.62 (1.79-3.82)* | 6.11E-07 |
|  | 70-75 | 1.31 (0.98-1.74) | 6.82E-02 |
|  | 75-80 | 1.17 (0.99-1.38) | 6.71E-02 |
|  | 80-85 | 1.09 (0.97-1.22) | 1.37E-01 |
|  | 85-90 | 0.98 (0.88-1.09) | 6.97E-01 |
|  | ≥90 | 0.95 (0.84-1.07) | 4.27E-01 |
| Atrial fibrillation | 65-70 | 1.49 (1.06-2.09)* | 2.25E-02 |
|  | 70-75 | 1.10 (0.88-1.38) | 3.85E-01 |
|  | 75-80 | 1.25 (1.09-1.42)* | 1.25E-03 |
|  | 80-85 | 1.05 (0.95-1.17) | 3.62E-01 |
|  | 85-90 | 1.16 (1.06-1.29)* | 2.39E-03 |
|  | ≥90 | 1.05 (0.93-1.20) | 4.19E-01 |

Hazard Ratio from model 6 (HR6) determined with Cox regression analyses using age as time scale adjusted for age at study entry and sex. *P-value <0.05.

**Table S6** Association of risk factors with mortality per age group

| Risk factor | Age group | HR (95% CI) | P-value |
| --- | --- | --- | --- |
| Hypertension | 65-70 | 1.55 (1.46-1.64)* | <1.00E-16 |
|  | 70-75 | 1.58 (1.49-1.67)* | <1.00E-16 |
|  | 75-80 | 1.53 (1.45-1.62)* | <1.00E-16 |
|  | 80-85 | 1.43 (1.36-1.52)* | <1.00E-16 |
|  | 85-90 | 1.53 (1.43-1.64)* | <1.00E-16 |
|  | ≥90 | 1.32 (1.24-1.41)* | 2.22E-16 |
| Diabetes Mellitus | 65-70 | 1.64 (1.54-1.75)* | <1.00E-16 |
|  | 70-75 | 1.60 (1.51-1.69)* | <1.00E-16 |
|  | 75-80 | 1.55 (1.47-1.63)* | <1.00E-16 |
|  | 80-85 | 1.43 (1.36-1.49)* | <1.00E-16 |
|  | 85-90 | 1.38 (1.32-1.45)* | <1.00E-16 |
|  | ≥90 | 1.28 (1.21-1.35)* | <1.00E-16 |
| Dyslipidemia | 65-70 | 1.33 (1.25-1.40)* | <1.00E-16 |
|  | 70-75 | 1.20 (1.14-1.26)* | 1.97E-12 |
|  | 75-80 | 1.1 (1.05-1.15)* | 5.11E-05 |
|  | 80-85 | 1.03 (0.99-1.08) | 1.27E-01 |
|  | 85-90 | 1.05 (1.01-1.10)* | 1.87E-02 |
|  | ≥90 | 0.97 (0.92-1.02) | 1.94E-01 |
| Stroke | 65-70 | 2.54 (2.28-2.84)* | <1.00E-16 |
|  | 70-75 | 2.33 (2.12-2.55)* | <1.00E-16 |
|  | 75-80 | 1.98 (1.84-2.14)* | <1.00E-16 |
|  | 80-85 | 1.80 (1.69-1.92)* | <1.00E-16 |
|  | 85-90 | 1.51 (1.41-1.61)* | <1.00E-16 |
|  | ≥90 | 1.35 (1.27-1.45)* | <1.00E-16 |
| Myocardial infarction | 65-70 | 1.73 (1.56-1.92)* | <1.00E-16 |
|  | 70-75 | 1.79 (1.64-1.95)* | <1.00E-16 |
|  | 75-80 | 1.51 (1.40-1.63)* | <1.00E-16 |
|  | 80-85 | 1.56 (1.46-1.67)* | <1.00E-16 |
|  | 85-90 | 1.37 (1.27-1.47)* | <1.00E-16 |
|  | ≥90 | 1.18 (1.09-1.28)* | 4.38E-05 |
| Heart failure | 65-70 | 4.58 (4.11-5.11)* | <1.00E-16 |
|  | 70-75 | 4.0 (3.67-4.36)* | <1.00E-16 |
|  | 75-80 | 3.42 (3.20-3.65)* | <1.00E-16 |
|  | 80-85 | 2.71 (2.57-2.86)* | <1.00E-16 |
|  | 85-90 | 2.11 (2.00-2.22)* | <1.00E-16 |
|  | ≥90 | 1.73 (1.65-1.82)* | <1.00E-16 |
| Atrial fibrillation | 65-70 | 1.64 (1.45-1.84)* | 4.44E-16 |
|  | 70-75 | 1.58 (1.45-1.73)* | <1.00E-16 |
|  | 75-80 | 1.71 (1.59-1.83)* | <1.00E-16 |
|  | 80-85 | 1.51 (1.43-1.60)* | <1.00E-16 |
|  | 85-90 | 1.54 (1.46-1.62)* | <1.00E-16 |
|  | ≥90 | 1.32 (1.25-1.40)* | <1.00E-16 |

Analyses are adjusted for age at study entry and sex; *P-value <0.05.

**Figures**

**Fig. S1**  Explanation of limited and continuous follow-up time

**Fig. S2**  Follow-up (FU) time difference between individuals with and without a risk factor. Positive values mean a longer FU time in the individuals without a risk factor.

**Fig. S1** Explanation of limited and continuous follow-up time


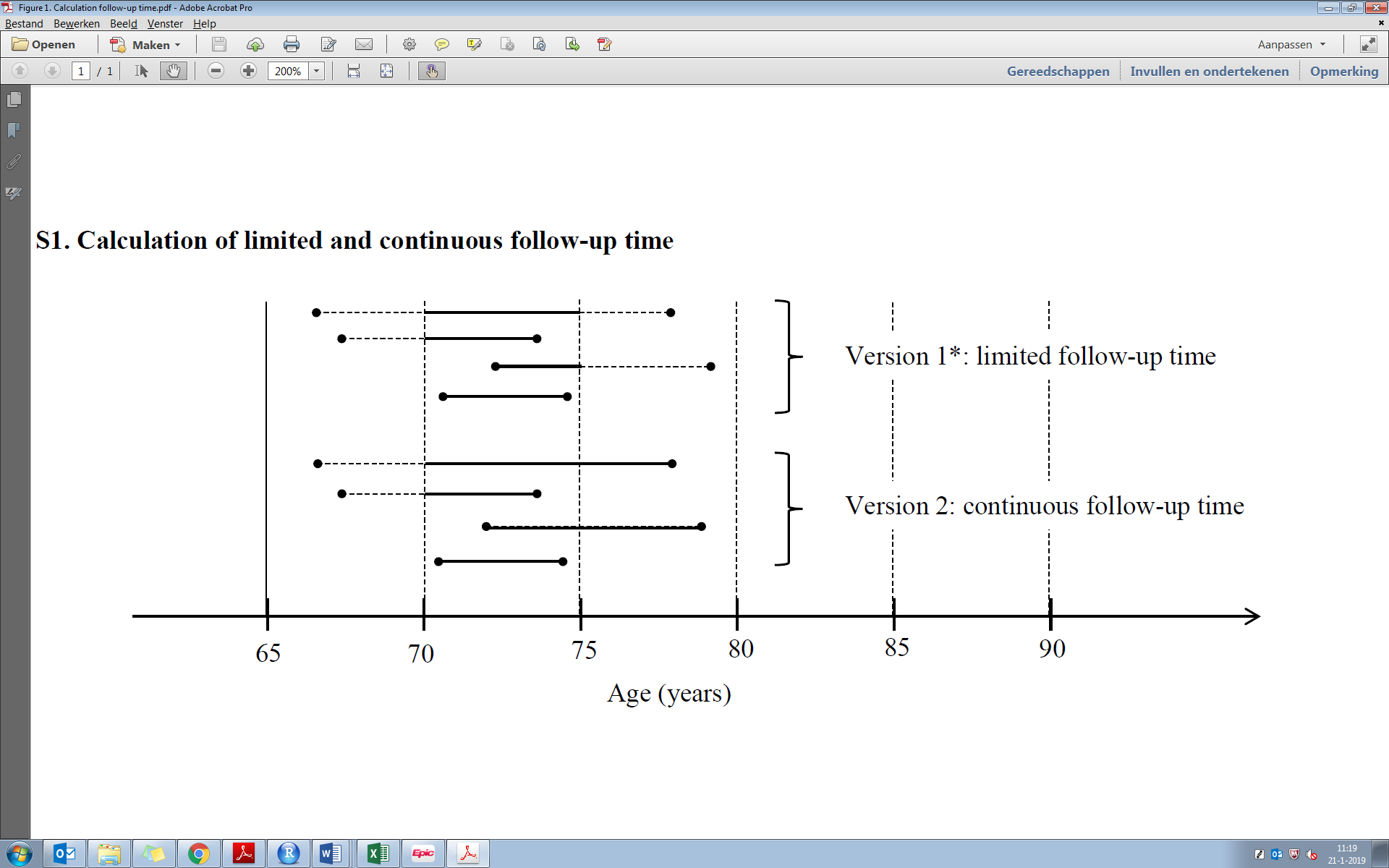


The dotted lines are not included in the follow-up time calculation for the 70-75 year old age group (used as example). *Version used in the main manuscript

**Fig. S2** Follow-up (FU) time difference between individuals with and without a risk factor. Positive values mean a longer FU time in the individuals without a risk factor.
